# Associations between trunk muscle morphology, strength and function in older adults

**DOI:** 10.1038/s41598-017-11116-0

**Published:** 2017-09-07

**Authors:** Behnaz Shahtahmassebi, Jeffrey J. Hebert, Mark D. Hecimovich, Timothy J. Fairchild

**Affiliations:** 1 School of Psychology and Exercise Science, Murdoch University, Perth, 6150 Australia; 2 Faculty of Kinesiology, University of New Brunswick, Fredericton, New Brunswick, Canada; 3 Division of Athletic Training, Iowa, University of Northern Iowa, Iowa, United States of America

## Abstract

Skeletal muscle plays an important role in performing activities of daily living. While the importance of limb musculature in performing these tasks is well established, less research has focused on the muscles of the trunk. The purpose of the current study therefore, was to examine the associations between functional ability and trunk musculature in sixty-four community living males and females aged 60 years and older. Univariate and multivariate analyses of the *a priori* hypotheses were performed and reported with correlation coefficients and unstandardized beta coefficients (*β*) respectively. The univariate analysis revealed significant correlations between trunk muscle size and functional ability (rectus abdominis: six-minute walk performance, chair stand test, sitting and rising test; lumbar multifidus: timed up and go) as well as trunk muscle strength and functional ability (trunk composite strength: six-minute walk performance, chair stand test, Berg balance performance, sitting and rising test). After controlling for covariates (age and BMI) in the multivariate analysis, higher composite trunk strength (*β* = 0.34) and rectus abdominis size (*β* = 0.33) were associated with better performance in the sitting and rising test. The importance of incorporating trunk muscle training into programs aimed at improving balance and mobility in older adults merits further exploration.

## Introduction

Age-related decreases in skeletal muscle size are accompanied by diminished muscle strength and function^1, 2^. In turn, these muscular and functional decrements are associated with a reduced quality of life^3^ and increased risk of falls^4^ among older adults. This increased risk of falls is a major health concern in terms of injury, disability and mortality, and is associated with an escalating socioeconomic burden^5^.

Previous studies investigating the relationship between muscle strength and functional outcomes in older adults have focused on peripheral musculature by examining handgrip strength and knee extensor strength^6^. However, more recent research has begun to focus on age-related changes in the trunk musculature^4, 7–9^ due to the important role of these muscles in performing activities of daily living, balance, mobility, and falls prevention in older adults^10–12^. A recent systematic review^12^ identified associations between trunk muscle strength/muscle attenuation (i.e., higher fat infiltration) and balance, functional ability, and risk of falls in older adults. In addition, the review identified a high level of heterogeneity between studies, and thus recommended further assessment of trunk muscle strength/composition, balance, and functional ability in older adults^12^.

Therefore, the primary aim of this study was to examine the associations between trunk muscle morphology (size), strength, and functional ability in older adults. We hypothesized that trunk muscle morphology and trunk muscle strength will be positively associated with functional ability in older adults. A secondary aim of this study was to investigate the association between trunk muscle morphology and strength in healthy older adults.

## Results

Sixty-four participants (38 female) with a mean (Standard Deviation; SD) age of 69.8 (7.5) years participated in this study. Descriptive data of the cohort are presented in Table 1.

**Table 1:** Descriptive characteristics of study cohort stratified by sex.

Characteristics	Outcomes		
	Total (n = 64)	Males (n = 26)	Females (n = 38)
% *population*	100%	40.6%	59.4%
*Age (years)*	69.8 (7.5)	68.8 (7.6)	70.4 (7.4)
*Body mass index (kg/m* ^2^)	27.3 (4.7)	27.9 (3.5)	27.0 (5.4)
*History of falls over past 1*2 *months*			
No falls	82%	92%	74%
Falls	18%	8%	26%
*Self-reported physical activity*			
Not very active (rarely leaves house)	3.1%	0%	5.3%
Moderately active (1-2 training sessions/week)	53.1%	57.7%	50.0%
Very active (≥3 training sessions/week)	43.8%	42.3%	44.7%
*Right total lateral abdominal muscles*, *cm*	1.6 (0.4)	1.9 (0.5)	1.49 (0.3)
*Left total lateral abdominal muscles*, *cm*	1.6 (0.39)	1.8 (0.4)	1.4 (0.3)
*Total lateral abdominal muscles (mean right/left)*, *cm*	1.6 (0.4)	1.8 (0.4)	1.4 (0.3)
*Rectus abdominis*, *cm* ^2^	4.1 (1.4)	5.3 (1.2)	3.2 (0.7)
*Lumbar multifidus L4/L5*, *cm*	3.1 (0.5)	3.3 (0.4)	3.0 (0.4)
*Lumbar multifidus L5/S1*, *cm*	3.0 (0.5)	3.2 (0.5)	2.9 (0.5)
*Composite trunk muscle size*, *cm*	8.5 (1.2)	9.2 (1.1)	8.1 (1.0)
*Trunk flexion strength*, *N*	125.0 (50.9)	166.3 (40.1)	96.8 (36.1)
*Trunk extension strength*, *N*	89.4 (44.9)	123.9 (41.5)	65.7 (29.4)
*Trunk right lateral flexion strength*, *N*	65.7 (29.6)	81.3 (33.4)	54.9 (21.2)
*Trunk left lateral flexion strength*, *N*	57.3 (26.0)	72.6 (26.8)	46.9 (19.8)
*Trunk lateral flexion strength (mean right/left)*, *N*	61.5 (26.5)	76.9 (28.2)	50.9 (19.4)
*Composite trunk strength*, *N*	337.5 (124.5)	444.2 (94.0)	264.5 (83.4)
*Six Minute Walk Test*, *m*	559.8 (87.9)	595.6 (86.3)	535.3 (81.4)
*30-Second Chair Stand Test*, *reps*	16.2 (4.45)	17.3 (5.2)	15.5 (3.6)
*Sitting and Rising Test*, *points*	5.7 (2.15)	6.5 (1.5)	5.1 (2.3)
*Berg Balance Scale*	52.0 (4.52)	52.7 (4.6)	51.6 (4.4)
*Timed Up and Go Test*, *sec*	7.4 (1.92)	7.2 (2.3)	7.5 (1.6)

Values are presented as mean (standard deviation; SD) or as total number and percentages.

*cm* (muscle thickness in centimeters), *cm* ^2^ (muscle cross sectional area in square centimeters), *L4/L5* lumbar spinal level at L4 and L5, *L5/S1* lumbar spinal level at L5 and S1, *reps* number of repetitions.

### Univariate Analysis

The univariate analysis between trunk muscle morphology and functional outcome measures (Table 2) revealed that a larger Rectus Abdominis (RA) cross sectional area (CSA) was associated with better six-minute walk time (6MWT; *r* = 0.27, *p* = 0.029), 30 second chair stand test (CST; *r* = 0.33, *p* = 0.007) performance and sitting and rising test (SRT (*r* = 0.29, *p* = 0.018) performance. While the thickness of LM-L5/S1 was positively correlated with TUG (*r* = 0.26, *p* = 0.037). The univariate associations between trunk muscle strength and functional outcomes (Table 3) revealed greater trunk extension strength was associated with better 6MWT (*r* = 0.35, *p* = 0.004), SRT (*r* = 0.38, *p* = 0.002) and BBS (*r* = 0.25, *p* = 0.042) outcomes; while lateral flexion strength was associated with better performance in the 6MWT (*r* = 0.33, *p* = 0.007), CST (*r* = 0.32, *p* = 0.010), SRT (*r* = 0.40, *p* = 0.001) and the BBS (*r* = 0.32, *p* = 0.007). Composite trunk strength was associated with better performance in the 6MWT (*r* = 0.35, *p* = 0.004), CST (*r* = 0.30, *p* = 0.016), SRT (*r* = 0.40, *p* = 0.001) and the BBS (*r* = 0.29, *p* = 0.017).

**Table 2:** Univariate analysis of associations between functional measures, descriptive characteristics (age, sex and BMI) and trunk muscle morphology.

	Age, y	Sex	BMI (kg/m^2^)	Rectus abdominis, cm^2^	Lumbar multifidus, cm		Total lateral abdominal muscles, cm			Composite trunk muscle size, cm
				CSA	L4/L5	L5/S1	Right	Left	Mean	
Six Minute Walk Test, m	**−0.67**	0.33	−0.20	**0.27**	−0.05	−0.10	0.23	0.16	0.21	0.06
	**(<0.001)**	(0.006)	(0.101)	**(0.029)**	(0.682)	(0.431)	(0.057)	(0.195)	(0.093)	(0.616)
30-Second Chair Stand Test, reps	**−0.48**	0.20	−0.12	**0.33**	−0.22	−0.22	0.23	0.15	0.20	−0.07
	**(<0.001)**	(0.107)	(0.321)	**(0.007)**	(0.076)	(0.071)	(0.062)	(0.227)	(0.106)	(0.558)
Sitting and Rising Test, points	**−0.59**	**0.32**	**−0.33**	**0.29**	−0.14	−0.20	0.20	0.15	0.18	−0.02
	**(<0.001)**	**(0.010)**	**(0.009)**	**(0.018)**	(0.266)	(0.104)	(0.109)	(0.229)	(0.143)	(0.848)
Berg Balance Scale	**−0.71**	0.12	−0.13	0.20	−0.19	−0.21	0.23	0.18	0.21	−0.04
	**(<0.001)**	(0.341)	(0.272)	(0.105)	(0.118)	(0.091)	(0.067)	(0.141)	(0.085)	(0.699)
Timed Up and Go Test, s	**0.75**	−0.08	0.10	−0.14	0.24	**0.26**	−0.17	−0.16	−0.17	0.12
	**(<0.001)**	(0.512)	(0.431)	(0.248)	(0.055)	**(0.037)**	(0.169)	(0.184)	(0.162)	(0.342)
Age, y	—	—	—	**−0.28**	0.08	0.14	**−0.25**	−0.21	−0.24	−0.02
				**(0.023)**	(0.527)	(0.244)	**(0.042)**	(0.087)	(0.051)	(0.819)
Sex	—	—	—	**0.73**	**0.29**	0.20	**0.46**	**0.47**	**0.48**	**0.46**
				**(<0.001)**	**(0.020)**	(0.101)	**(<0.001)**	**(<0.001)**	**(<0.001)**	**(<0.001)**
BMI (kg/m^2^)	—	—	—	**0.37**	**0.41**	**0.40**	**0.44**	**0.51**	**0.49**	**0.52**
				**(0.002)**	**(0.001)**	**(0.001)**	**(<0.001)**	**(<0.001)**	**(<0.001)**	**(<0.001)**

Values are presented are Pearson correlation coefficients, except sex was presented by point biserial correlation (exact *p* values).

Bolded estimates are statistically significant at *p* ≤ 0.05 and *p* ≤ 0.01.

*BMI* body mass index, *cm* (muscle thickness in centimeters), *cm* ^2^ (muscle cross sectional area in square centimeters), *CSA* cross sectional area, *L4/L5* lumbar spinal level L4/L5, *L5/S1* lumbar spinal level L5/S1, *Composite trunk muscle size* comprised the thickness of bilateral lateral abdominal muscles, rectus abdominis, lumbar multifidus L4/L5, lumbar multifidus L4/L5, *n* number of participants, *reps* repetitions, *s* seconds.

**Table 3:** Univariate analysis of associations between functional measures, descriptive characteristics (age, sex and BMI) and trunk muscle strength.

	Trunk strength, N		Trunk Lateral Flexion strength, N			Composite trunk strength, N
	Flexion	Extension	Right	Left	Mean	
Six Minute Walk Test, m	0.23	**0.35**	**0.29**	**0.28**	**0.33**	**0.35**
	(0.059)	**(0.004)**	**(0.018)**	**(0.025)**	**(0.007)**	**(0.004)**
30-Second Chair Stand Test, reps	0.19	0.22	**0.30**	**0.32**	**0.32**	**0.30**
	(0.128)	(0.072)	**(0.016)**	**(0.009)**	**(0.010)**	**(0.016)**
Sitting and Rising Test, points	0.22	**0.38**	**0.40**	**0.33**	**0.40**	**0.40**
	(0.076)	**(0.002)**	**(0.001)**	**(0.007)**	**(0.001)**	**(0.001)**
Berg Balance Scale	0.17	**0.25**	**0.33**	**0.27**	**0.32**	**0.29**
	(0.175)	(0.042)	(0.007)	(0.030)	(0.007)	(0.017)
Timed Up and Go Test, s	−0.14	−0.14	−0.17	−0.18	−0.19	−0.19
	(0.248)	(0.268)	(0.169)	(0.148)	(0.127)	(0.132)
Age, y	−0.24	−0.20	**−0.27**	−0.24	**−0.27**	**−0.28**
	(0.056)	(0.111)	**(0.027)**	(0.057)	**(0.019)**	**(0.022)**
Sex	**0.67**	**0.64**	**0.44**	**0.48**	**0.48**	**0.71**
	**(<0.001)**	**(<0.001)**	**(<0.001)**	**(<0.001)**	**(<0.001)**	**(<0.001)**
BMI (kg/m^2^)	**0.47**	0.004	0.06	0.08	0.07	0.22
	**(<0.001)**	(0.974)	(0.622)	(0.499)	(0.509)	(0.070)

Values are presented are Pearson correlation coefficients, except sex was presented by point biserial correlation (exact *p* values).

Bolded estimates are statistically significant at *p* ≤ 0.05 and *p ≤ *0.01.

*BMI* body mass index, *Composite trunk strength* comprised trunk strength flexion, extension and lateral flexion (the average of right and left).

The univariate associations between trunk muscle morphology and strength are presented in Table 4. The major findings were that a larger TLAM thickness (All p ≤ 0.007) and a larger CSA of the RA (All p < 0.001) were consistently associated with increased trunk flexion, trunk extension, trunk lateral flexion and composite trunk muscle strength.

**Table 4:** Univariate analysis of associations between trunk muscle morphology and strength

	Rectus abdominis, cm^2^	Lumbar multifidus, cm		Total lateral abdominal muscles, cm			Composite trunk muscle size, cm
	CSA	L4/L5	L5/S1	Right	Left	Mean	
Trunk flexion strength, N	**0.80**	**0.27**	0.21	**0.68**	**0.68**	**0.70**	**0.54**
	**(<0.001)**	**(0.026)**	(0.086)	**(<0.001)**	**(<0.001)**	**(<0.001)**	**(<0.001)**
Trunk extension strength, N	**0.51**	0.20	0.13	**0.40**	**0.33**	**0.38**	**0.33**
	**(<0.001)**	(0.106)	(0.284)	**(0.001)**	**(0.007)**	**(0.002)**	**(0.006)**
Trunk right lateral flexion strength, N	**0.44**	−0.01	−0.060	**0.38**	**0.41**	**0.41**	0.17
	**(<0.001)**	(0.884)	(0.637)	**(0.002)**	**(0.001)**	**(0.001)**	(0.164)
Trunk left lateral flexion strength, N	**0.44**	−0.00	−0.04	**0.37**	**0.38**	**0.39**	0.18
	**(<0.001)**	(0.988)	(0.700)	**(0.002)**	**(0.002)**	**(0.001)**	(0.152)
Trunk lateral flexion strength (mean right/left), N	**0.46**	−0.01	−0.05	**0.40**	**0.41**	**0.42**	0.18
	**(<0.001)**	(0.929)	(0.651)	**(0.001)**	**(0.001)**	**(0.001)**	(0.138)
Composite trunk strength, N	**0.71**	0.18	0.11	**0.59**	**0.58**	**0.60**	**0.42**
	**(<0.001)**	(0.148)	(0.374)	**(<0.001)**	**(<0.001)**	**(<0.001)**	**(<0.001)**

Values are presented are Pearson correlation coefficients (exact *p* values).

Bolded estimates are statistically significant at *p* ≤ 0.05 and *p* ≤ 0.01.

*BMI* body mass index, *cm* (muscle thickness in centimeters), *cm* ^2^ (muscle cross sectional area in square centimeters), *Composite trunk muscle size* comprised the thickness of bilateral lateral abdominal muscles, rectus abdominis, lumbar multifidus L4/L5, lumbar multifidus L4/L5, *Composite trunk strength* comprised trunk strength flexion, extension and lateral flexion (the average of right and left), *CSA* cross sectional area, *n* number of participants, *N* newton.

### Multivariate Analysis

The multivariate analysis between muscle morphology and functional measures (Table 5) showed that after controlling for covariates, the CSA of the RA was associated with the 6MWT (*β* = −0.27; *p* = 0.050) and the SRT (*β* = 0.33; *p* < 0.001) outcome. After controlling for covariates, there was a significant association between composite trunk strength and the performance in the SRT (*β* = 0.34; *p* < 0.001).

**Table 5:** Multiple linear regression analysis of the relationship between trunk muscle morphology and strength with functional measures

Variable		Adjusted *R* ^2^	*R* ^2^ change significance	Standardized *β* coefficient	*β* coefficient significance
**Trunk muscle morphology and functional measures**					
***Six Minute Walk Test***, ***m***					
Model	Age	0.53	<0.001	−0.70	<0.001
	Sex			0.46	<0.001
	Rectus abdominis CSA, cm^2^			−0.27	0.050
***30-Second Chair Stand Test***, ***sec***					
Model	Age	0.25	<0.001	−0.42	<0.001
	Rectus abdominis CSA, cm^2^			0.21	0.064
***Sitting and Rising Test***, ***points***					
Model	Age	0.60	<0.001	−0.57	<0.001
	BMI			−0.52	<0.001
	Rectus abdominis CSA, cm^2^			0.33	<0.001
***Timed Up and Go Test***, ***cm***					
Model	Age	0.58	<0.001	0.58	<0.001
	Lumbar multifidus L5/S1, cm			0.15	0.068
**Trunk muscle strength and functional measures**					
***Six Minute Walk Test***, ***m***					
Model	Age	0.508	<0.001	−0.63	<0.001
	Sex			0.21	0.063
	Trunk extension strength, N			0.08	0.449
***30-Second Chair Stand Test***, ***reps***					
Model	Age	0.25	<0.001	−0.42	<0.001
	Trunk lateral flexion strength (mean right/left), N			0.21	0.066
***Sitting and Rising Test***, ***points***					
Model	Age	0.60	<0.001	−0.56	<0.001
	BMI			−0.47	<0.001
	Composite trunk strength, N			0.34	<0.001
***Berg Balance Scale***					
Model	Age	0.52	<0.001	−0.67	<0.001
	Trunk right lateral flexion strength, N			0.14	0.112

The levels of significance are set at *p* ≤ 0.05 and *p* ≤ 0.01.

*BMI* body mass index, *cm* (muscle thickness in centimeters), *cm* ^2^ (muscle cross sectional area in square centimeters), *Composite trunk strength* comprised trunk strength flexion, extension and lateral flexion (the average of right and left), *n* number of participants, *N* newton, *reps* repetitions.

The multivariate analysis exploring the relationship between trunk muscle morphology and strength (Table 6) demonstrated significant associations between trunk flexion strength and the CSA of the RA (*β* = 0.45; *p* = 0.001) along with the TLAM thickness (*β* = 0.29; *p* = 0.003). After controlling for sex and age, the CSA of the RA was associated with composite trunk strength (*β* = 0.34; *p* = 0.007).

**Table 6:** Multiple linear regression analysis of the relationship between trunk muscle morphology and strength.

Variable		Adjusted *R* ^2^	*R* ^*2*^ change significance	Standardized *β* coefficient	*β* coefficient significance
**Trunk flexion strength**, **N**					
Model 1	Rectus abdominis CSA, cm^2^	0.68	<0.001	0.60	<0.001
	Total lateral abdominal muscles (mean right/left), cm			0.28	0.005
Model 2	Sex	0.70	<0.001	0.19	0.060
	Rectus abdominis CSA, cm^2^			0.45	0.001
	Total lateral abdominal muscles (mean right/left), cm			0.29	0.003
**Trunk extension strength**, **N**					
Model	Sex	0.40	<0.001	0.56	<0.001
	Rectus abdominis CSA, cm^2^			0.10	0.469
**Trunk right lateral flexion strength**, **N**					
Model	Age	0.18	<0.001	−0.19	0.096
	Sex			0.29	0.082
	Rectus abdominis CSA, cm^2^			0.17	0.326
**Trunk left lateral flexion strength**, **N**					
Model	Sex	0.22	<0.001	0.35	0.035
	Rectus abdominis CSA, cm^2^			0.18	0.264
**Trunk lateral flexion strength (mean right/left)**, **N**					
Model	Sex	0.25	<0.001	0.35	0.032
	Age			−0.19	0.096
	Rectus abdominis CSA, cm^2^			0.14	0.383
**Composite trunk strength**, **N**					
Model 1	Rectus abdominis CSA, cm^2^	0.52	<0.001	0.56	<0.001
	Total lateral abdominal muscles (mean right/left), cm			0.21	0.079
Model 2	Age	0.58	<0.001	−0.14	0.100
	Sex			0.44	0.001
	Rectus abdominis CSA, cm^2^			0.34	0.007

The levels of significance are set at *p* ≤ 0.05 and *p* ≤ 0.01.

*BMI* body mass index, *cm* (muscle thickness in centimeters), *cm* ^2^ (muscle cross sectional area in square centimeters), *Composite trunk strength* comprised trunk strength flexion, extension and lateral flexion (the average of right and left), *CSA* cross sectional area, *n* number of participants, *N* newton.

## Discussion

The most important outcomes of this study were: i) univariate analyses revealed small-moderate positive correlations between trunk muscle morphology, strength and functional outcome measures; ii) after controlling for covariates (age, sex/BMI) the CSA of the RA demonstrated significant associations with functional outcomes (6MWT and SRT scores), while composite trunk strength was significantly associated with performance in the SRT; iii) measures of trunk strength appeared to demonstrate stronger and more consistent univariate associations with functional ability than measures of trunk morphology, although this was not demonstrated in the multivariate analysis. The findings of the current study align with our stated hypotheses, although the relationship between trunk muscle morphology and function were not as consistent as the relationships between trunk muscle strength and function. Specifically, composite trunk muscle size was not associated with any functional outcomes, which is in contrast to composite trunk strength, which was associated with four out of five (6MWT, CST, SRT, BBS) functional tasks. In addition to the above main findings, age, sex, and/or BMI had strong influences on performance in various functional tasks.

The univariate analysis between trunk muscle morphology and function revealed only small to moderate relationships between the CSA of the RA and three functional outcomes (6MWT, CST, SRT); while LM thickness at the L5/S1 demonstrated an association with the TUG task (Table 2). Importantly however, the composite trunk muscle size demonstrated no significant associations with functional outcomes. After adjusting for covariates in the multiple linear regression models, only the CSA of the RA (*β* = 0.33; Table 5) was retained in the model (*R* ^2^ = 0.60) for the SRT outcome. The ability to sit and rise from the floor unassisted (measured with the Sitting and Rising Test; SRT) has been identified as a predictor of all-cause mortality and is an important functional measure in older adults^13^, wherein each one-point increase in the SRT is associated with a 21% reduction in all-cause mortality^13^. It is noteworthy that BMI (*β* = −0.52) and age (*β* = −0.57) were the covariates retained in the model, suggesting younger participants with lower BMI performed better during this task. To the authors’ knowledge, only one previous study^11^ has explored the relationship between trunk muscle morphology (lumbar paraspinal, lateral abdominal, and rectus abdominis muscles) and performance of functional tasks in healthy older adults (70–79 y.o.). Similar to the findings of the present study, Hicks *et al*. found that after controlling for covariates (age, sex, race, height, total body fat and thigh muscle composition) the average trunk muscle area was not associated with performance on the Health ABC Physical Performance Battery.

The univariate analysis between strength and functional ability demonstrated consistent positive associations (Table 3) although only composite trunk strength (*β* = 0.34; Table 5) was retained in the final multivariate model (*R* ^2^ = 0.60) for the SRT, along with age (*β* = −0.56) and BMI (*β* = −0.47). The associations between trunk muscle strength and functional tasks (BBS and TUG) have previously been explored in two studies^7, 10^. Suri *et al*.^10^ demonstrated that isometric trunk extension strength was moderately correlated with the BBS (*r* = 0.41, *p* < 0.05) which is consistent with our findings (*r* = 0.25, *p* < 0.05). Of note, Suri *et al*.^10^ suggested the variance explained by trunk extension endurance was either equivalent to or exceeded the variance explained by limb strength across all three adopted measures of performance (Berg Balance Scale; Unipedal Stance Test; Short Physical Performance Battery). The association between measures of trunk muscle strength and performance on the TUG has previously been examined by Granacher *et al*.^7^ and in accord with the findings of the current study (All *p* > 0.1) they found no significant associations. The difference in the associatoins between the TUG and the BBS with trunk muscle strength are unclear and while speculative, they may in part be due to the TUG requiring multiple dimensions of balance and mobility while the BBS comprises a number of static tasks which may be more reliant on trunk stabilisation. It is noteworthy that the univariate associations between functional tasks and trunk muscle strength were not greater for the derived composite score (Table 5).

In addition to the findings above, our study demonstrated strong positive correlations between trunk muscle morphology (size) and trunk muscle strength (Table 4). Specifically, RA CSA (*β* = 0.45; Table 6) was retained in the multivariate model (*R* ^2^ = 0.70) for trunk flexion strength, along with sex. TLAM thickness (*β* = 0.29; Table 6) was retained in the final multivariate model (*R* ^2^ = 0.70) for trunk flexion strength, along with sex. RA CSA (*β* = 0.34; Table 6) was retained in the model (*R* ^2^ = 0.58) for composite trunk strength, along with age and sex. The results of the current study are in line with the findings of Andersen *et al*.^14^, who examined the association between trunk muscle cross-sectional area (CT; attenuation) and trunk strength in older adults (≥65 y.o.). Andersen *et al*.^14^ reported that trunk muscle attenuation was associated with absolute strength, however, the association between trunk muscle cross-sectional area and absolute strength was larger across all studied muscles (anterior abdominal muscles; posterior abdominal muscles; paraspinal muscles; combined). These findings appear consistent with the general role abdominal muscles play in providing stability in the trunk region^15^ rather than acting as a prime mover. The finding that age and sex strongly correlate with trunk muscle morphology and strength (Tables 2 and 3) is also consistent with previous studies^14, 16, 17^.

It is noteworthy that the univariate analysis revealed more consistent associations between trunk muscle strength and functional performance (Table 3) than compared to trunk muscle morphology and functional performance (Table 2). However, these associations did not translate to the multivariate analyses, where the descriptive characteristics and most notably age (Table 5) played the dominant role in explaining the variance in outcome measures. It is surprising that the CSA of the RA demonstrated more consistent associations with functional measures than other muscle groups such as the LM, since the RA is not a primary muscle involved in these activities. Trunk muscle (psoas muscle) sarcopenia has previously been identified as an objective measure of frailty^18^ and has been found to strongly correlate with post-surgical mortality (liver transplant^19^; adrenocortical carcinoma^20^; aortic aneurysm^18^). While speculative, this may suggest the associations between the RA CSA and functional measures in this study may be due to the RA CSA providing a measure of frailty in this population, rather than suggesting a direct involvement of the RA in the performance of these tasks. This speculation lends support from the fact that the CSA of the RA was retained in the model for performance of SRT, which is a task which has previously been identified as being a predictor of all-cause mortality^13^.

The study presented herein had several strengths, including i) comprehensive examination of the associations between trunk muscle morphology, strength, and functional ability across multiple domains in healthy older adults; ii) the maximum isometric trunk torque (Nm) data being normalized to trunk height (cm), allowing comparison across study participants^21, 22^. However, several factors may limit the interpretation and application of findings from this study. While the number of participants (n = 64) was sufficient to conduct the analyses, the number of predictor variables in the models (i.e., multivariate linear regression) were restricted. Secondly, the participants in this study were healthy and moderately active older adults. Therefore, the results may not generalize to other populations such as individuals with mobility or balance limitations). Specifically, the study cohort performed well in the BBS (52.0 ± 4.5) and TUG (7.4 ± 1.9 sec), wherein cut-offs of 45^23^ and less than 10 seconds^24^ are regarded as established criterion to identify older adults with high risk of falls and good physical mobility respectively. Accordingly, only 18% of the cohort in this study reported a fall in the previous 12-month period (Table 1). As with others studies, the results herein relate specifically to the testing methodology adopted; namely trunk muscle morphology, trunk muscle strength and functional ability. While each outcome measure was assessed across multiple domains, the outcomes are unlikely to represent all components of trunk muscle morphology, strength, mobility, and balance. Further, while ultrasound imaging is a reliable and valid assessment of trunk muscle morphology, it may not accurately capture important intrinsic characteristics in muscle quality (e.g. intermuscular fat infiltration) that accompany aging. Additionally, ultrasound imaging may be complicated by excessive adipose tissue (i.e., individuals who are obese) and this occurred in two individuals in the cohort, and the who presented a challenge for capturing the total muscle belly. Finally, this study utilized a cross-sectional study design, and thus the findings of this study cannot be used to infer causation.

The extant literature assessing the relationships between physical function and age-related declines in muscle morphology and strength are largely focused on measures of peripheral musculature^1, 6, 25^; with only limited studies exploring these associations with trunk musculature^7, 10, 11^. The current study builds on these previous studies and provides a comprehensive account of the relationships between trunk muscle morphology (size), strength, and functional ability in a cohort of healthy, older participants. Specifically, our findings revealed significant associations between trunk muscle morphology and trunk muscle strength with performance of functional tasks in older adults. The extent to which these associations are due to either direct involvement of this musculature in task performance, or due to an indirect association (i.e., trunk muscle morphology and/or strength as a surrogate marker of frailty) remains to be fully established. Considering that the trunk musculature is responsive to exercise training^26^, the cross-sectional findings herein provide additional support for the incorporation of trunk muscle training into exercise programs aimed at improving functional performance in older adults. However, interventional research targeting these muscles is required to validate the importance of trunk muscle training to improve functional performance in an aged cohort.

## Methods

This cross-sectional study examined the associations between trunk muscle morphology, strength, and functional ability (functional outcome measures categorized into either functional mobility or balance outcome measures) in healthy older adults. The study used baseline data of participants enrolled into a Randomized Controlled Trial (ACTRN12613001176752) between February 2014 and October 2015. The Murdoch University Human Research Ethics Committee approved the study protocol (No. 2013/140), and all experiments were performed in accordance with relevant guidelines and regulations. All participants provided written informed consent prior to enrolment.

### Participants

Men and women aged 60 years and older were recruited from the local community and aged care facilities. Participants were excluded if they i) had previously undergone lumbar spine surgery, ii) had any medical condition(s), or were taking prescribed medication that precluded safe participation in an exercise intervention, or iii) were unable to communicate in English.

### Anthropometric and demographic characteristics

Body weight was measured using a digital scale (Scales Plus, Perth, WA, Australia) and height (standing and seated) using a wall-mounted stadiometer (Surgical Medical Supplies Pty Ltd, Adelaide, SA, Australia). Seated height (the length of the trunk) was assessed using the distance from the highest point on the head to the sitting surface and was measured using the wall-mounted stadiometer. Physical activity levels and demographic data were collected through self-report.

### Functional mobility and balance

Functional mobility was assessed using the Six Minute Walk Test (6MWT^27^), the 30-second Chair Stand Test (CST^28^), and the Sitting and Rising Test (SRT^13^). The scores of the 6MWT were reported as the distance (m) walked during the 6 minutes while the CST results were based on the number of successful repetitions in 30 sec^28^. The SRT measures the individual’s ability to sit and rise unassisted from the floor with partial scores assigned from the two required actions of sitting (5 points) and rising (5 points) and a final composite SRT score then reported (ranging from 0 to 10^13^). Balance was assessed using the Berg Balance Scale (BBS^23^) and the Timed Up and Go Test (TUG^24^). The BBS comprises 14 items of static and dynamic balance tasks, and scores are presented as a summed score with a maximum of 56 points. The TUG results were presented in time (seconds) to complete the task.

### Trunk muscle morphology

**Figure 1:**
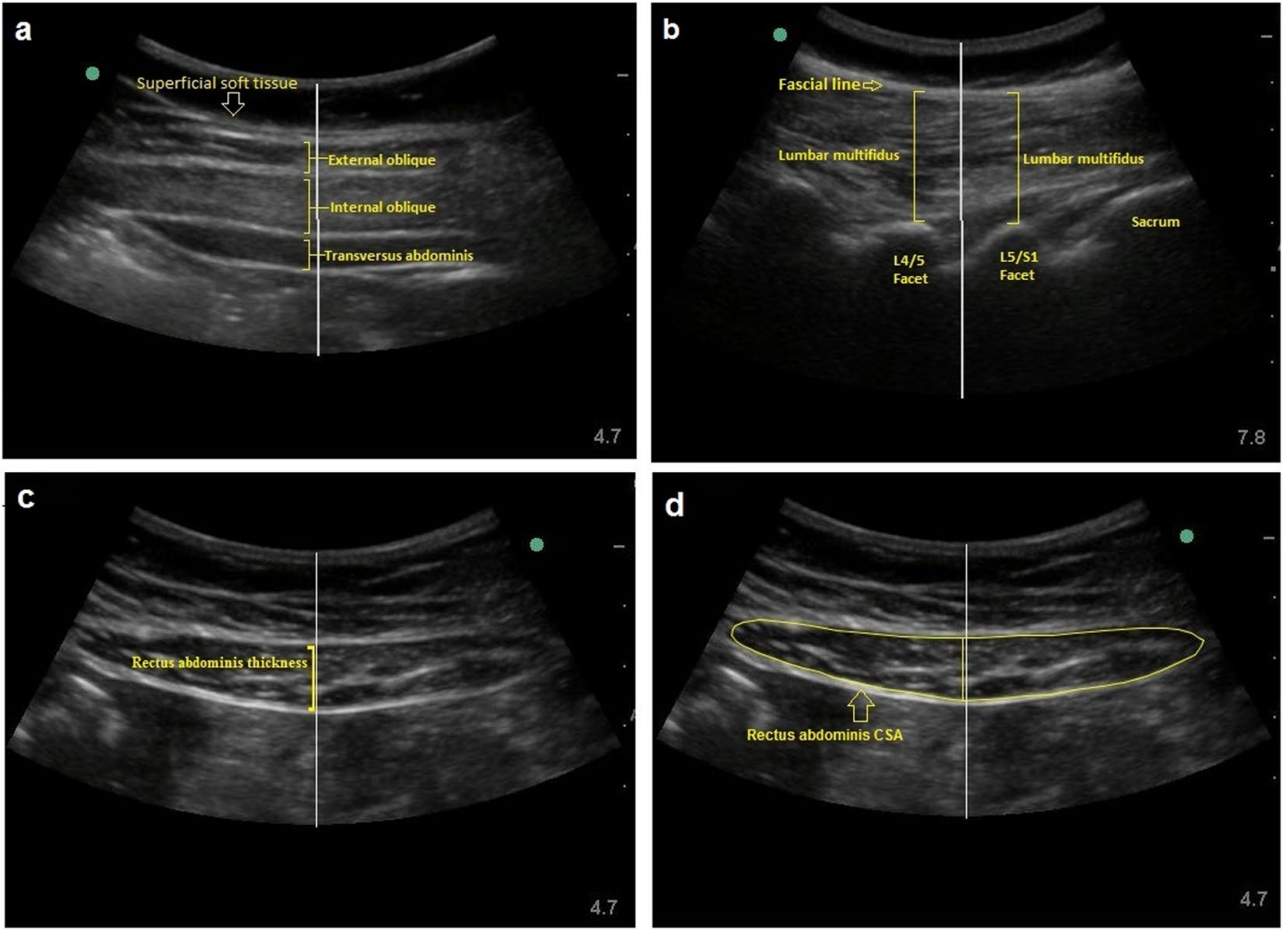
Ultrasound images of the lateral, anterior abdominal and posterior trunk muscles at rest. (**a**) Thickness measurements of lateral abdominal muscles were made between the superficial and deep borders of the external oblique, internal oblique, and transversus abdominis muscles in the middle of the muscle belly; (**b**) Thickness measurements of lumbar multifidus were made between the posterior-most portion of the L4/L5 and L5/S1 facet joints, and the plane between the muscle and subcutaneous tissue; (**c**) Measurement of the rectus abdominis muscle thickness was obtained between the deep and superficial borders of the rectus abdominis muscle; (**d**) Measurement of the cross-sectional area of the rectus abdominis muscle was obtained by tracing the interior border of the rectus abdominis muscle.

An ultrasound unit (SonoSite™, Bothell, WA, USA) with a 60 mm broadband curved array (5-2 MHz) was used to measure the size of the rectus abdominis (RA), internal oblique (IO), external oblique (EO), transversus abdominis (TrA) and lumbar multifidus (LM) muscles (Fig. 1). Previous studies using ultrasound imaging to measure trunk muscle size in older adults have demonstrated high inter-rater and intra-rater reliability (ICC ≥ 0.86)^29, 30^. Images of the lumbar multifidus (LM) were obtained at the L4-5 level (L4/L5) with the participant in the prone position using methods described in previous studies^31^. Rectus abdominis (RA) thickness and cross-sectional area (CSA), as well as transversus abdominis (TrA), internal oblique (IO) and external oblique (EO) thickness was measured with participants in the supine, hook-lying position. The images were captured with the middle of the muscle belly centered in the field of view and at the end of a normal exhalation to control for the influence of respiration^31^. For acquisition of the RA, the inferior border of the transducer was placed immediately above the umbilicus and moved laterally from the midline until the muscle cross-section was centered in the image^32^. Image acquisition was performed three times bilaterally and exported for offline analysis using Image J (SonoSite™, Bothell, WA, USA). All measures were averaged across the three repetitions to reduce measurement error^31^.

A composite trunk muscle size variable was created for the total lateral abdominal muscles (TLAM) by summing the thickness of TrA, IO, and EO. A second composite trunk muscle size variable was created from the TLAM (left and right) thickness, rectus abdominis, and lumbar multifidus (average of right and left) at lumbar spinal level L4/L5 (L4/L5) and L5/S1 (L5/S1).

### Trunk muscle strength

Maximal isometric strength in trunk flexion, extension, and lateral flexion was assessed using an Isokinetic dynamometer (Humac NORM, Computer Sports Medicine, Stoughton, MA, USA) with the trunk extension–flexion (TEF) modular component; which has been reported to be a reliable and valid method for measuring trunk muscle strength^33, 34^. The participant was positioned and fastened into the machine as per manufacturer instructions and previous study description^35^. The strength testing was performed in the same order each time: trunk flexion, extension and then lateral flexion (right, left). Prior to testing, participants performed a standardized warm-up consisting of one set (10 repetitions) of range of motion exercises and up to five practice trials. For maximal efforts, contractions were held for 3 seconds and the peak torque from two attempts recorded. A familiarization trial preceded each measure and the participant rested for 45 seconds between each repetition^36^. Verbal encouragement was provided during each effort. Maximum isometric trunk torque (Nm) data was normalized by adjusting for trunk height (cm) and converting the peak torque to maximum force (N) [Maximum force = Peak torque/Moment arm (trunk height)]. Therefore, all data on trunk muscle strength are presented as maximum force. A composite trunk strength score was calculated by summing the maximum forces from flexion, extension, lateral flexion right and lateral flexion left.

### Data analysis

All data management and statistical analyses were performed using IBM SPSS version 21.0 softwaref. The relationships between trunk muscle morphology, trunk muscle strength and functional outcome measures, were examined with univariate and multivariate analyses. We first explored these relations with Pearson’s correlation coefficients (*r*) for continuous independent variables or point-biserial coefficients for dichotomous independent variables. Where independent variables demonstrated significant correlations (*p* ≤ 0.05) with the outcome measures, these were then included in separate multivariate linear regression models. When only one muscle predictor was identified at the univariate step, it was force entered into the model along with significant demographic covariates. When more than one muscle predictor was identified by the univariate analysis, they were entered into a hierarchical model. The muscle predictor explaining the greatest variance in the outcome measures was then included in step two with the significant demographic covariates. If more than three variables qualified for entry (e.g., a combination of two demographic variables and two potential predictors), we selected the strongest demographic variable only. Standardized beta coefficients (*β*) were generated for each of the variables retained in the final model and adjusted *R* ^2^ values were calculated at each step. The level of significance was set at *p* ≤ 0.05.

### Data availability

The data that support the findings of this study are available from the corresponding author (TJF) on request.
